# ASO Author Reflections: Decoding Racial Differences in Colon Cancer Transcriptomes

**DOI:** 10.1245/s10434-024-16682-y

**Published:** 2024-12-07

**Authors:** Mohamad El Moheb, Allan Tsung

**Affiliations:** Department of Surgery, University of Virginia Health, Charlottesville, VA USA

Despite significant advancements in colon cancer treatment, Black patients continue to experience worse outcomes compared with white patients across all disease stages, even when socioeconomic and access-to-care factors are accounted for.^1^ This persistent disparity suggests the influence of underlying biological differences contributing to more aggressive tumor behavior in Black patients. To investigate these differences at the molecular level, this study aimed to comprehensively examine the stage-specific gene expression profiles of Black and white patients with colon cancer. Identifying these transcriptomic signatures is critical, as current precision oncology approaches are largely based on data from white populations,^2^ which may overlook the unique, stage-specific gene expression profiles that influence treatment responses and progression mechanisms in Black patients. Our study lays the groundwork for developing more tailored therapies that may help bridge the survival gap between these groups.^3^

Utilizing data from the National Cancer Database, we confirmed that Black patients with colon cancer have worse overall survival compared with white patients. After stratifying patients by disease stage (localized, regional, and metastatic), 561 genes were differentially expressed between Black and white patients. These differences highlighted significant biological variations, as evidenced by high clustering in tumor expression profiles based on race. Notably, the differentially expressed genes varied by disease stage, indicating that transcriptomic—and thus biological—differences are dynamic and evolve with disease progression. These genes can be broadly categorized into seven functional groups as they relate to cancer: angiogenesis and tumor migration, immune response and inflammation, metabolic processes, signal transduction and cellular communication, cellular adhesion and structure, transcription regulation and development, and other functions. Our gene set enrichment analysis highlighted the biologic relevance of these genes, revealing that Black patients with localized disease show suppressed immune-related pathways, specifically in the IL-17 and cytokine–cytokine receptor interaction pathways. This suggests a suppressed tumor immune microenvironment in early-stage disease, potentially facilitating tumor growth and immune evasion, contributing to more aggressive disease in Black patients.

We further identified five genes—*RAMACL*, *POLR2J3*, *POLR2J2*, *MUC16*, and *PRSS21*—that were consistently differentially expressed across all stages and that exhibited high discriminatory power in classifying patients by race (area under the curve: 0.863–0.880). These genes may represent a stage-independent transcriptomic signature for Black patients, potentially linked to increased tumor aggressiveness within the context of evolving stage-specific differences.

Further research is needed to build on our findings and address gaps in understanding racial disparities in colon cancer. For instance, the observed IL-17 suppression in Black patients could indicate an enhanced response to immune checkpoint inhibitors, as IL-17 suppression has been shown to reduce protumorigenic inflammation and boost CD8^+^ cytotoxic T lymphocyte activity.^4^ Testing this hypothesis could provide critical insights into tailored immunotherapy approaches for Black patients. More broadly, our study provides a foundational blueprint of the tumor transcriptional profile specific to Black patients with colon cancer, moving beyond the traditional focus on white patient profiles. This blueprint opens pathways for the development of targeted therapies that account for racial differences, potentially bridging the survival gap and offering a more personalized approach to treatment for Black patients. By improving confidence in treatment responses, these advancements could also increase Black patient enrollment in randomized trials, fostering more inclusive and representative clinical research. Additionally, advanced techniques such as single-cell RNA sequencing and spatial transcriptomics offer opportunities for deeper, cell-specific insights into tumor biology, elucidating immune pathway interactions, such as IL-17 and cytokine pathways, and further refining race-specific treatments.
